# Posterior placenta accreta spectrum: unraveling distinctive clinical features and diagnostic challenges

**DOI:** 10.7150/ijms.127179

**Published:** 2026-02-26

**Authors:** Yating Wu, Kuifang Shen, Caihong Hu, Jianling Wei, Jingrui Huang, Chenlin Pei

**Affiliations:** 1 Department of Obstetrics, Xiangya Hospital Central South University, Changsha 410008, China.; 2 Hunan Engineering Research Center of Early Life Development and Disease Prevention, Changsha 410008, China.

**Keywords:** prenatal diagnosis, placenta accreta spectrum, ultrasound

## Abstract

**Background:**

Posterior placenta accreta spectrum (PAS) represents a diagnostically challenging subtype of PAS, which has long been overlooked in existing literature due to its relatively low incidence and nonspecific clinical manifestations.

**Methods:**

A comprehensive literature search was conducted in PubMed, Web of Science, and other databases, focusing on studies related to the diagnosis and management of posterior PAS. This review systematically analyzes the risk factors, prenatal diagnostic characteristics, and pregnancy outcomes of posterior PAS, with a focus on clarifying the characteristic differences between posterior PAS and PAS involving other uterine locations.

**Results:**

Both ultrasound (US) and magnetic resonance imaging (MRI) have limited diagnostic sensitivity for posterior placenta accreta spectrum (PAS): the sensitivity of US ranges from 56.4% to 62%, while the application rate of MRI signs for posterior PAS is 73.5%, and both of which are 20-30% lower than those for anterior PAS. Irregular retroplacental clear zone on US and cervical varices on MRI are prominent diagnostic indicators for the condition. Notably, compared with anterior PAS, posterior PAS is characterized by shallower myometrial invasion and a more favorable prognosis.

**Conclusions:**

Posterior PAS has unique diagnostic and clinical features that distinguish it from anterior PAS. Enhanced recognition of its imaging characteristics and targeted management strategies are crucial to improve maternal outcomes. This review fills the literature gap by systematically summarizing the latest evidence on posterior PAS, providing a reference for clinical practice and future research.

## Background

Placenta accreta spectrum (PAS) disorders refer to a condition in which, after delivery of the fetus, the placenta fails to detach partially or completely from the uterine wall^1^. The prevailing hypothesis suggests that a defect in the endometrium-myometrial interface—typically at the site of a prior hysterotomy—leads to impaired decidualization in the affected uterine area. This pathological change facilitates excessive extravillous trophoblast infiltration, allowing villous tissue to invade deeply into the myometrium, including its vascular network, and in some cases, even into adjacent pelvic organs^2^, ^3^. Based on the depth of trophoblast invasion, pathologists classify PAS into three subtypes: (1) superficial placenta accreta (also called placenta creta, vera, or adherenta); (2) placenta increta; (3) placenta percreta^2^, ^3^. Theoretically, any primary uterine anomaly or secondary damage to the uterine wall structure can lead to PAS disorders, including the invasive forms^2^-^4^. Epidemiological studies demonstrate that PAS is strongly associated with prior cesarean sections (CS)^5^-^8^, with most cases involving the anterior uterine wall, which has been the primary focus of current research^9^, ^10^.A 2025 multicenter study indicated that the prenatal ultrasound diagnostic rate for posterior PAS is only 62%, 20% lower than that for anterior PAS, and this discrepancy highlights the particularity and challenges in the prenatal diagnosis of posterior PAS^11^. However, posterior PAS exhibits distinct clinical features. Morgan *et al.* also reported that posteriorly located PAS is associated with delayed diagnosis, higher surgical complication rates, increased use of assisted reproductive technology (ART), and fewer prior cesarean deliveries compared to anterior PAS^12^. Tinari *et al.* found that the incidence of PAS in women with a posterior placenta was 4.8%^13^. Despite its clinical significance, research on posterior PAS remains scarce, particularly regarding its incidence, prenatal screening efficacy, and perinatal outcomes^14^. This review synthesizes current evidence on posterior PAS, systematically analyzing its risk factors, antenatal diagnostic approaches, and pregnancy outcomes. Special emphasis is placed on contrasting its clinical characteristics with those of anterior PAS, aiming to enhance diagnostic accuracy and optimize management strategies for affected patients.

## Risk factors

The prevailing hypothesis suggests that a defect in the endometrium-myometrial interface—typically secondary to uterine scarring—disrupts normal decidualization, permitting abnormal invasion of placental anchoring villi and trophoblasts into the myometrium. This mechanism has been clinically validated^3^. PAS disorders are exceptionally rare in primigravid women without underlying uterine pathology^4^, ^15^. However, any surgical procedure compromising endometrial integrity is associated with PAS, including: cesarean delivery, uterine curettage, hysteroscopic surgery, myomectomy, fractional curettage, and *in vitro* fertilization (IVF)^2^, ^3^, ^16^. Notably, Tinari *et al.* (2021) reported that 72.6% of posterior PAS cases involved concurrent placenta previa and prior uterine surgery (predominantly cesarean section). None of the cases had a history of termination of pregnancy (TOP) or intrauterine device (IUD) use, whereas 38.05% were linked to IVF^13^. The majority of risk factors identified in women with posterior PAS are similar to those reported in anterior PAS, including placenta previa, prior cesarean section, or a history of uterine surgery^11^. However, compared with anterior PAS, ART, lower numbers of prior cesarean deliveries and the absence of prior cesarean section have a stronger association with posterior PAS^12^, ^14^. In contrast, factors such as maternal age, bodymass index (BMI), geographic region, occupation, antepartum hemorrhage, emergency delivery, multiple gestation, and fetal position have no significant influence on placental location^14^. In summary, for patients with an anterior placenta, focus should be placed on assessing the accumulation of prior cesarean section history; for those with a posterior placenta, the evaluation of ART conception history and various uterine surgical histories should be strengthened to improve the pertinence and accuracy of prenatal diagnosis.

## Prenatal diagnosis

### Ultrasound examination

**Figure 1:**
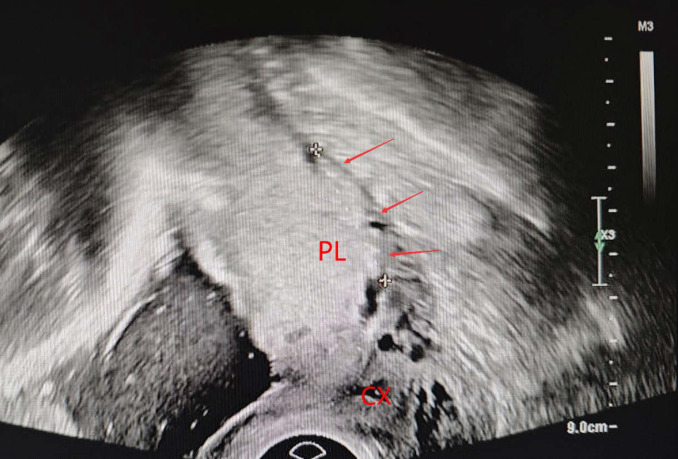
Loss or irregularity of the hypoechoic area between the uterus and posterior placenta (arrow). PL, placenta. CX, cervix

**Figure 2:**
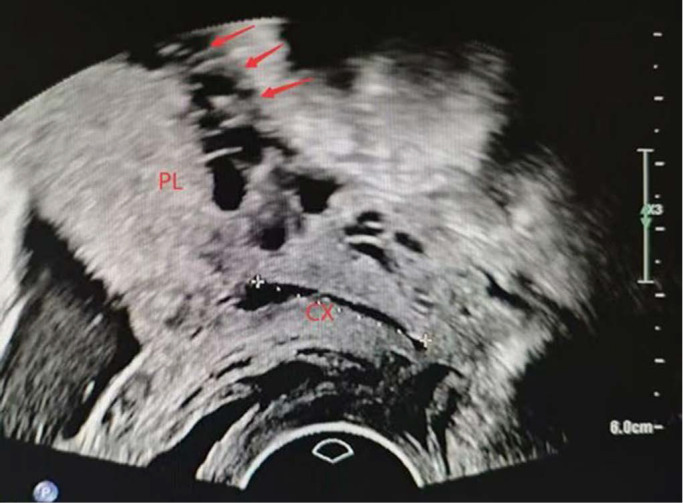
placental lacunae (arrow). PL, placenta. CX, cervix

**Figure 3:**
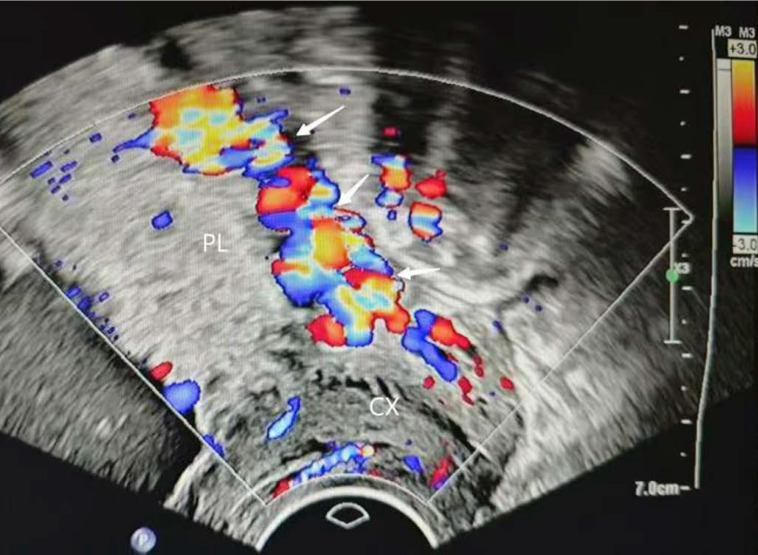
irregular intraplacental vascularization of posterior placenta(arrow) PL, placenta. CX, cervix.

Ultrasound (US) and magnetic resonance imaging (MRI) have shown high diagnostic performance in the detection of PAS^17^-^19^. Ultrasound serves as the primary diagnostic tool for PAS; however, current clinical sonographic criteria are primarily validated for anterior PAS cases complicated by placenta previa. When the placenta invades the posterior uterine wall, the diagnostic accuracy of prenatal ultrasound in detecting characteristic signs remains uncertain^17^, ^18^, ^20^. Dellapiana *et al.* reported that the application of standard sonographic criteria results in a low antenatal detection rate for posterior PAS^21^. For optimal ultrasound diagnosis of posterior PAS, the recommended gestational window for initial screening is between 25 and 28 weeks. This timing is advantageous because: the uterine wall and placental architecture are more clearly visualized at this stage, the amniotic fluid-to-fetal size ratio is more favorable, and the posterior implantation site becomes more accessible for evaluation^22^. The ultrasound markers of PAS include: (1) Loss or irregularity of the hypoechoic area between the uterus and placenta, the 'retroplacental clear zone' (Figure 1), (2) Myometrial thickness <1mm, (3)placental lacunae with high velocity flow (>15cm/s) (Figure 2), (4)Thinning or interruption of the uterine serosa-bladder wall interface, (5) Placental bulge, (6) Exophytic mass,(7) Subplacental and/or uterovesical hypervascularity, (8) Loss of vascular arch parallel to the basal plate and irregular intraplacental vascularization (Figure 3)^3^, ^23^. After excluding the two bladder line-dependent criteria, the following four key sonographic features were consistently observed in the posterior placental region: loss or irregularity of the hypoechoic area between the uterus and placenta, myometrial thickness <1mm, placental lacunae with high velocity flow (>15cm/s), and Loss of vascular arch parallel to the basal plate and irregular intraplacental vascularization^22^. Many authors have adopted a two-criteria system in their articles: PAS is diagnosed when there are two or more ultrasonic signs present^22^, ^24^. The two-criteria system has a high sensitivity (60.0%), specificity (98.9%), and positive predictive value (85.7%). Moreover, when using a single criterion or the optimal criterion, there is no improvement in the maternal outcomes of true positive and false negative cases, which confirms the reliability of the two-criteria system^22^. A retrospective study has found that the sensitivity of ultrasound in detecting anterior PAS is as high as 81 - 93%^18^. Other studies have also validated this discrepancy. The ADoPAD (Antenatal Diagnosis of Placental Attachment Disorders) Study Group reported that prenatal ultrasound detected 92% of all anterior PAS cases, which was significantly higher than the 62% detection rate for posterior PAS^11^. The detection rates of PAS in anterior and posterior placentas were reported by Pilloni *et al.* as 89.7% and 50% respectively, under the application of the two - criteria system^24^. However, in a recent study, after excluding patients with placenta previa, only 30% of posterior placenta PAS cases were diagnosed through prenatal ultrasound screening. This suggests that compared with anterior PAS, ultrasound examination has lower sensitivity for posterior PAS^21^. An independent evaluation of the sensitivity of individual ultrasound signs for pathologically confirmed posterior and anterior PAS was conducted. The sensitivities of retroplacental lacunae, vascular congestion, myometrial thinning, and absence of hypoechoic areas in detecting posterior wall PAS ranged from 24% to 42%. However, multiple studies have demonstrated that the absence/irregularity of the retroplacental clear zone shows the highest sensitivity and negative predictive value for posterior placenta accreta - comparable to or even exceeding that of the dual-criteria system. These findings confirm the diagnostic importance of this specific criterion for posterior placenta accreta, while other criteria exhibit significantly lower sensitivities compared to the dual-criteria system^22^, ^24^. This divergence primarily stems from hemodynamic differences, as the posterior uterine wall's lower segment demonstrates greater vascularity compared to the anterior wall. Sonographically, the retroplacental clear zone shows better echogenic contrast with posterior placental positioning than with anterior implantation. Consequently, when placental invasion disrupts the retroplacental clear zone's integrity, sonographic abnormalities become more detectable on the posterior uterine wall. This diagnostic advantage is most evident between 26 and 28 weeks of gestation^24^. In summary, ultrasound demonstrates suboptimal sensitivity for detecting posterior PAS. As the first-line imaging modality, its diagnostic performance is limited by three key factors: (1) operator-dependent variability, (2) reduced efficacy in obese patients, and (3) poor detection of posterior placental invasion. These limitations collectively compromise both the accuracy and clinical utility of ultrasound for posterior PAS diagnosis. Furthermore, current research on posterior PAS remains constrained by small sample sizes, highlighting the need for larger-scale studies to validate existing findings^22^.

### Ultrasound scoring scale

To better quantify PAS severity and predict surgical risks prenatally while optimizing perioperative management for invasive placentation, researchers worldwide have developed various ultrasound assessment systems. Among these, the "Ultrasound Scoring Scale for Placental Invasion" developed by Professor Zhao's team has been clinically implemented. This validated scoring system evaluates nine critical sonographic parameters: placental distance to cervical os, placental thickness, retroplacental hypoechogenicity, bladder serosa integrity, placental lacunar patterns, basal vascularity, cervical sinusoidal dilation, cervical morphological alterations, and prior cesarean history. Each parameter is systematically scored according to predefined criteria, with the cumulative total serving as a numerical indicator of invasion severity. Importantly, the "placental location" parameter in this scale specifically measures the inferior placental margin's proximity to the internal os, which differs fundamentally from anatomical classifications based on implantation site (anterior/posterior wall). Notably, the scoring algorithm does not incorporate placental implantation location as a variable. Additionally, it should be noted that the validation study population primarily consisted of patients with both placenta previa and prior cesarean deliveries, which may limit the scale's applicability to other clinical contexts^25^. In 2019, an ultrasound staging system was established for PAS in women with placenta previa, adopting a 0, 1, 2, 3 grading approach. This system was evaluated in relation to surgical outcomes and placental invasion, demonstrating congruence with the clinical PAS staging system proposed by the International Federation of Gynecology and Obstetrics (FIGO)^26^. Current ultrasound scoring systems have been primarily validated in populations with both placenta previa and prior cesarean delivery. Despite this specific validation context, these scoring systems are routinely applied to posterior wall PAS cases, even though they demonstrate consistently lower sensitivity scores for this anatomical variant. Most importantly, current diagnostic guidelines fail to provide position-adjusted ultrasound criteria or customized scoring systems for different placental locations, representing a substantial limitation in standardized diagnostic approaches for varying implantation sites.

### Magnetic resonance imaging examination

Although magnetic resonance imaging (MRI) and US demonstrate comparable predictive accuracy for PAS, MRI is preferentially recommended for evaluating posterior wall PAS. MRI offers superior capabilities in precisely mapping potential sites of placental abnormalities, especially in cases involving elevated BMI or posterior placental implantation. This advantage stems from MRI's wider field of view, which enables high-resolution placental assessment, along with its greater reproducibility across studies compared to ultrasound^27^. The diagnostic accuracy of MRI in PAS evaluation is significantly dependent on gestational timing. Current evidence supports 28-32 weeks gestation as the optimal imaging window, as later uterine enlargement and myometrial stretching reduce detection sensitivity for focal myometrial thinning at the placental-serosal interface. Additionally, physiological placental changes in late gestation - manifesting as heterogeneous signal intensity and infarction-like changes on MRI - may mimic pathological features of PAS, further complicating diagnosis. These factors collectively diminish the reliability of invasion pattern assessment in advanced pregnancy. Nevertheless, clinical guidelines recommend individualized MRI timing based on specific clinical scenarios^17^, ^28^. Ultrasound has limited prenatal diagnostic efficacy for posterior PAS. A systematic review by Tinari *et al.* showed that only 56.4% of posterior PAS cases could be detected by prenatal ultrasound. In contrast, MRI exhibits superior diagnostic performance, with 73.5% of confirmed cases identifiable via prenatal MRI^13^. A targeted study by Budorick *et al.* further corroborates the advantages of MRI: Among the 10 cases pathologically confirmed, ultrasonic examinations yielded 3 false positive results, whereas MRI examinations demonstrated perfect specificity with no false positive findings. Consequently, they recommended that when dealing with posterior wall placentas associated with PAS risk factors and inconclusive ultrasonic manifestations, MRI should be prioritized as a supplementary diagnostic modality^29^. The 2018 FIGO guidelines indicate that for cases of posterior placenta with suspected penetrating placenta accreta, MRI can be considered. The uteroplacental interface of the posterior placenta may not be comprehensively evaluated by ultrasound due to the increased depth and partial obstruction by the fetus. However, MRI is not affected by these factors^29^, ^30^. On T2-weighted images, several imaging features have been proposed as indicators for PAS. These include intraplacental dark bands, which may suggest altered placental-myometrial interfaces; placental heterogeneity, indicative of variable pathological changes such as invasion or hemorrhage within the placenta; abnormal intraplacental vessels, characterized by increased vascularity, tortuosity, or irregular patterns; focal exophytic masses, representing placental tissue extending beyond the normal placental contour; and placental elevations, often resulting from local tissue proliferation or edema at the site of invasion.

Regarding placental bed vascular changes, excessive uterine serosal vessels, the "vesical vascular" sign (abnormal vascular engorgement near the bladder wall), and the "parametrial vascular" sign (abnormal vascularity in the parauterine region) have been associated with abnormal placental invasion. Additionally, interruption of the myometrium directly demonstrates placental penetration into the uterine muscle, while interruption of the bladder wall with a characteristic "tenting" appearance indicates advanced PAS with bladder involvement^31^. To date, no studies have specifically explored whether there are differences in the magnetic MRI signs of PAS between anterior and posterior placental locations. However, conducting targeted research on the variations in MRI manifestations corresponding to different placental positions—with a particular focus on the anterior and posterior walls—may further enhance clinicians' diagnostic and therapeutic awareness, facilitate the accurate interpretation of MRI features related to posterior placentas, optimize the diagnostic efficacy for posterior placental PAS, and provide more targeted reference support for the clinical management of posterior PAS^32^. In 2017, Hiroki Ishibashi and his colleagues utilized these specific indicators to predict the coexistence of placenta previa and posterior uterine wall placenta accreta. The sensitivity of these indicators was found to be 0%, while the specificity reached 97.4%. The positive predictive value was 0%, and the negative predictive value was 96.2%^33^. Currently, the imaging manifestations revealed by MRI also pose significant challenges for the prenatal diagnosis of posterior PAS. Maurea and colleagues have established that placental elevation serves as an independent MRI predictor for PAS disorders^34^. However, a study by Tao Lu *et al.* indicated that the imaging manifestation of placental bulge is closely associated with placental location and is less common in posterior placentas. They proposed that when the placenta is located on the posterior uterine wall, it may be more susceptible to compression by the maternal spine or sacrum, resulting in a lower incidence of placental bulge in such cases. In contrast, when the placenta is situated on the anterior uterine wall, the placental bulge is not compressed and thus more easily identifiable^32^. Ishibashi put forward a novel imaging marker to quantify the severity of cervical varices, aiming to improve the diagnostic accuracy of PAS in cases of placenta previa. To quantify the cervical varices on the posterior aspect of the cervix, specific measurements are taken on T2-weighted sagittal MRI slices. These measurements include the shortest distances from the most posterior cervical varices to the placental decidual surface (designated as distance A), the placental amniotic surface (distance B), and the internal os of the cervix. Subsequently, the ratio of distance A to distance B (the A/B ratio) is computed. Moreover, a combined receiver operating characteristic (ROC) curve is constructed for the A/B ratio. In addition, a separate combined ROC curve is created by incorporating placental hyperplasia with the A/B ratio. The area under the curve (AUC) of this combined ROC curve reaches an impressive 0.96. When the cut - off value of the A/B ratio is set at 0.18, the diagnostic performance is remarkable. It attains a sensitivity of 100%, indicating that all true - positive cases can be correctly identified. The specificity stands at 91.0%, suggesting a high ability to accurately classify negative cases. Overall, this innovative approach substantially boosts the sensitivity of prenatal MRI screening for PAS in placenta previa^33^. Notably, cervical varices have been established as a characteristic associated with placenta previa^35^. However, it remains uncertain whether the diagnostic accuracy for posterior PAS would improve upon the exclusion of placenta previa - related risk factors.

### MRI Scoring Systems

Currently, research on MRI placenta accreta scoring systems is relatively scarce both in domestic and international medical communities. In 2016, Ueno *et al.* developed the first MRI-based predictive model for placenta accreta. This model employs a Likert scale to assign scores to six MRI characteristics, namely, dark bands on T2-weighted images, abnormal vascularity within the placenta, placental protuberance, heterogeneous placental signals, myometrial thinning, and the placental protrusion sign. The scoring scale ranges from 1 to 5. Studies have indicated that this model demonstrates a high diagnostic efficacy for placenta previa complicated by invasive PAS disorders^36^. Delli Pizzi *et al.* implemented a systematic evaluation of eight MRI features associated with PAS using a 5-point Likert scale. Their assessment expanded upon prior work by incorporating two additional characteristics— placental lacunae and parauterine invasion—beyond the previously established six features. Building on this foundation, the research team successfully developed in 2019 a dedicated radiological scoring system. This model serves as a standardized, objective tool for assessing PAS severity based exclusively on MRI findings^37^. In 2022, the radiology team at China Medical University meticulously curated a comprehensive set of 10 MRI biomarkers and 1 clinical parameter. The MRI-derived features encompassed the number of prior cesarean deliveries, placental localization, placental/uterine convexity, placental signal inhomogeneity, T2-weighted hypointense placental bands, intrapartum vascular anomalies, abnormal angioarchitecture of the placental bed, disruption of the T2 hypointense uteroplacental interface, transmural invasion of the urinary bladder, penetrating placenta increta, along with focal myometrial thinning and discontinuity. Comprising 11 distinct scoring components, this system employed a tiered severity - based scoring paradigm, assigning 0, 1, or 2 points to each parameter. Cumulatively, the total score serves as a quantifiable metric for stratifying the severity of pernicious placenta previa, facilitating standardized clinical assessment and risk stratification^38^. Notably, the placental location parameter aligns with the corresponding criterion in the previously discussed ultrasound scoring system. An intriguing area for exploration lies in whether integrating the specific location of placenta accreta into the scoring model could enhance its predictive efficacy. Currently, existing MRI - based scoring systems predominantly focus on severe anterior PAS cases accompanied by placenta previa.

Ultrasound is generally the primary tool for diagnosing and assessing the risk of PAS. In contrast, MRI is utilized to confirm the diagnosis or serve as an adjuvant diagnostic method when the ultrasound evaluation of posterior PAS yields inconclusive results^39^. The scientific literature contains only two published studies that have systematically compared the sensitivity of ultrasound versus MRI for posterior PAS diagnosis^13^, ^22^. As shown in Table 1,^13^, ^22^, all four ultrasound signs investigated for posterior PAS diagnosis exhibit low sensitivity (all below 60%). Among these, the absence of the retroplacental clear zone is the most sensitive indicator, while bladder-related findings are rarely observed in posterior wall PAS cases.

When it comes to the description of the sensitivity of MRI signs for the posterior uterine wall, two relevant literatures could be retrieved^13^, ^32^. As shown in Table 2,^13^, ^32^, the sensitivity of these signs is uniformly low, with the sensitivity of the bladder bulging sign showing the lowest sensitivity. Notably, bladder-associated signs on B-mode ultrasound were not detected in any cases, which is hypothesized to be related to the relative anatomical positions of the uterus and bladder.

## Stages of placenta accreta and Pregnancy outcomes

Liu Hong and her colleagues classified placental implantations at different locations into three groups in their article: the anterior uterine wall group (where more than 50% of the placenta is attached to the anterior uterine wall), the posterior uterine wall group (where more than 50% of the placenta is attached to the posterior uterine wall), and the non-central placenta group (cases other than those attached to the anterior and posterior uterine walls). The placental locations on the anterior uterine wall and non-central uterine wall have a more significant impact on the severity of PAS compared to that on the posterior uterine wall. The risk of invasive placenta accreta in the anterior uterine wall placenta group is 3.13 times higher than that in the posterior uterine wall placenta group, and the risk of invasive placenta in the non-central placenta PAS group is 1.90 times higher than that in the posterior uterine wall placenta group^14^. A 2021 meta-analysis focusing on posterior PAS revealed that roughly 78% of such cases exhibited placenta increta^13^. The ADoPAD Study Group further confirmed that the majority of pregnancies complicated by posterior PAS are characterized by placenta accreta, with the incidence of placenta percreta being significantly lower than that in anterior PAS (54%vs 10%)^11^. In 2024, Feng Xiaoling and co - authors demonstrated in their research that the anterior PAS group exhibited a significantly higher incidence of invasive PAS compared to both the posterior PAS group and the (lateral/basal) PAS group. Among cases with equivalent degrees of placental invasion, the anterior wall and (lateral/basal) PAS groups showed substantially higher prenatal diagnosis rates than the posterior PAS group^40^. Feng Xiaoling and colleagues compared delivery outcomes among three groups, finding that the posterior wall PAS group showed a significantly higher incidence of emergency cesarean section - approximately five times greater than the anterior wall PAS group (17.3% vs. 3.4%). Additionally, the mean gestational age at delivery for posterior wall PAS patients was 36.4±2.45 weeks, which was significantly earlier than the other groups. These findings were corroborated by Charis Bourgioti *et al.*, who similarly reported significantly higher emergency delivery rates in posterior PAS cases compared to both anterior PAS and combined anterior/posterior PAS groups^41^. This phenomenon may be attributed to delayed diagnosis of posterior wall PAS. The ADoPAD Study Group also confirmed that compared with patients with anterior PAS, those with posterior PAS had a lower rate of hysterectomy requirement (88.5%vs48%), while the rates of emergency CS and balloon tamponade were significantly higher. The lower risk of hysterectomy in patients with posterior PAS may be attributed to the lower incidence of placenta percreta in this subgroup^11^. According to the statistical analysis by Feng Xiaoling *et al.*, no significant differences were observed in perinatal outcomes between posterior wall PAS and PAS at other uterine locations, including estimated 24-hour blood loss, blood component transfusion requirements, abdominal/pelvic visceral injury rates, and ICU admission rates^40^. However, the research conducted by Charis Bourgioti and other scholars has demonstrated that the perinatal outcomes of PAS vary significantly depending on the placental attachment location. Specifically, the posterior PAS group has a lower probability of developing severe complications such as placental invasion into the uterine cavity with protrusion and bladder involvement. In comparison to cases of posterior PAS, patients with anterior PAS and PAS in other locations often experience more severe intraoperative bleeding. Additionally, the total duration of childbirth is significantly prolonged, and the length of stay in the intensive care unit (ICU) after surgery is also notably increased^41^. The team of Elizabeth A. Morgan found that compared with placenta accreta in other uterine wall locations, ureteral injury is the only complication that shows a increased incidence rate in surgeries for posterior placenta accreta^12^.

## Management

The management of PAS strictly adheres to the core principles of "precise assessment, individualized plan, and multidisciplinary team (MDT) collaboration", and differentiated management strategies for posterior PAS must be formulated based on its anatomical and clinical characteristics^42^. Due to the concealed anatomical location of posterior PAS, a combined diagnostic approach of ultrasound plus contrast-enhanced pelvic MRI is recommended. This approach further improves diagnostic accuracy by precisely clarifying the depth and scope of lesion invasion as well as the anatomical adjacency to the rectum and ureters, thereby reducing the rate of emergency surgery and the risk of severe complications such as intraoperative massive hemorrhage^11^, ^42^. Preoperative MDT collaboration is a key link in optimizing management, requiring the joint participation of obstetricians, anesthesiologists, transfusion specialists, interventional radiologists, and urologists to collectively complete risk stratification, blood transfusion preparation, and individualized surgical plan development. Furthermore, all cases are advised to undergo planned delivery at 34-36 weeks in specialized centers with dedicated PAS diagnosis and treatment qualifications to minimize the risk of adverse maternal and neonatal outcomes^42^. Compared with anterior PAS, posterior PAS is characterized by shallower placental invasion and a significantly lower hysterectomy rate^11^, ^40^, ^41^, therefore conservative surgeries such as focal resection plus uterine repair are preferred for patients with posterior PAS, while hysterectomy is only considered in cases of deep invasion or uncontrollable intraoperative massive hemorrhage. Conservative hemostatic measures are the first choice for hemostasis; techniques such as balloon tamponade and B-Lynch suture are recommended to effectively control bleeding, reduce unnecessary hysterectomy, and maximize the preservation of the patient's fertility^42^.

## Conclusion

There are significant heterogeneities in risk factors, diagnostic difficulty, clinical characteristics, and prognosis of PAS with different uterine attachment sites (anterior wall vs. posterior wall) (Table 3). Accurate differentiation and targeted management are crucial for improving maternal and infant outcomes.

In summary, compared with anterior PAS, posterior wall PAS is strongly associated with prior non-cesarean uterine procedures and assisted reproduction. Ultrasound/MRI demonstrate limited sensitivity, though irregular retroplacental clear zone on ultrasound and cervical varices on MRI aid diagnosis. These cases typically show shallow invasion and better outcomes than anterior PAS.

## Figures and Tables

**Table 1:** Range of Diagnostic Accuracy of Ultrasound signs for PAS in the Posterior Wall

ultrasound signs	Diagnostic accuracy
Loss of the clear zone	41.05-60%
Bladder wall interruption	16.64%-20%
Placental lacunae	38.95%-50%
Hypervascularity at the bladder wall interface	0%

**Table 2:** Range of Diagnostic Accuracy of MRI signs for PAS in the Posterior Wall

MRI signs	Diagnostic accuracy
Intra-placental dark bands	45.62-52.17%
Uterine bulging	0-19.57%
Bladder tenting	0-2.17%
Focal interruption of the myometrium	26.09-45.62%
Heterogenous signal intensity	26.09%-45.62%

**Table 3:** Key Differences Between Anterior and Posterior PAS

Comparison Item	Anterior PAS	Posterior PAS
Core Risk Factors	Previous cesarean section(12, 14)	Previous non-cesarean uterine procedures and ART(12, 14)
Prenatal Diagnosis Rate(US)	81-100%^11^, ^13^	56.4-62%^11^, ^13^
Prenatal Diagnosis Rate(MRI)	>90%^13^	73.5%^13^
depth of invasion	severe placental invasion^11^, ^14^, ^40^	placenta accreta^11^, ^14^, ^40^
hysterectomy rate	88.5%^11^	48%^11^

## Abbreviations

PAS: placenta accreta spectrum
US: ultrasound
MRI: magnetic resonance imaging
CS: cesarean section
ART: assisted reproductive technology
IVF: *in vitro* fertilization
TOP: termination of pregnancy
IUD: intrauterine device
BMI: body mass index
ADoPAD: Antenatal Diagnosis of Placental Attachment Disorders
FIGO: International Federation of Gynecology and Obstetrics
ROC: receiver operating characteristic
AUC: area under the curve
MDT: multidisciplinary team
